# Agarose spot migration assay to measure the chemoattractant potential of extracellular vesicles: applications in regenerative medicine and cancer metastasis

**DOI:** 10.1186/s12915-023-01729-5

**Published:** 2023-10-26

**Authors:** Marta Clos-Sansalvador, Marta Monguió-Tortajada, Ferran Grau-Leal, Vicenç Ruiz de Porras, Sergio G. Garcia, Marta Sanroque-Muñoz, Miriam Font-Morón, Marcella Franquesa, Francesc E. Borràs

**Affiliations:** 1 REMAR-IGTP Group, Germans Trias i Pujol Research Institute (IGTP) & Nephrology Department, University Hospital Germans Trias i Pujol (HUGTiP), Can Ruti Campus, Badalona, Barcelona, Catalonia 08916 Spain; 2 Department of Cell Biology, Physiology and Immunology, Universitat Autònoma de Barcelona (UAB), Bellaterra, Spain; 3 ICREC Research Program, Germans Trias i Pujol Research Institute (IGTP) & Cardiology Department, University Hospital Germans Trias i Pujol (HUGTiP), Can Ruti Campus, Badalona, Catalonia Spain; 4 RCPB Group, CARE Program, Germans Trias i Pujol Research Institute (IGTP); ProCURE Program, Catalan Institute of Oncology, Carretera de Can Ruti, Camí de Les Escoles S/N, Badalona, 08916 Spain; 5 CARE Program, Germans Trias i Pujol Research Institute (IGTP), Badalona, Spain; 6 Catalan Institute of Oncology, Badalona Applied Research Group in Oncology (B·ARGO), Badalona, Spain; 7 Department of Cell Biology, Physiology and Immunology, Universitat de Barcelona (UB), Barcelona, Spain

**Keywords:** Extracellular vesicles, Exosomes, Migration, Recruitment, Agarose, Cancer, Regenerative medicine, Function, Metastasis

## Abstract

**Background:**

The recruitment of effector cells is one of the novel functions described for extracellular vesicles (EVs) that needs further study. For instance, cell recruitment by mesenchymal stromal cell derived-EVs (MSC-EVs) is one of the features by which MSC-EVs may induce regeneration and ameliorate tissue injury. On the other hand, increasing evidence suggests that cancer EVs play an important role in the preparation of the pre-metastatic niche (PMN) by recruiting their primary tumour cells. Understanding and measuring the potential of MSC-EVs or cancer-EVs to induce cell migration and recruitment is essential for cell-free therapeutic approaches and/or for a better knowledge of cancer metastasis, respectively. In this context, classical in vitro migration assays do not completely mimic the potential situation by which EVs exert their chemotactic capacity.

**Results:**

We adapted an agarose spot migration assay as an in vitro system to evaluate the cell recruitment capacity of locally delivered or localized EVs. Cell migration was tracked for 12 h or 48 h, respectively. Thereafter, endpoint migration images and time-lapse videos were analysed to quantify several parameters aiming to determine the migration of cells to either MSC-EV or pro-metastatic EV. The number of cells contained inside the agarose spots, the migration distance, the area occupied by cells, the directionality of the cell movement, and the Euclidean distance were measured. This multi-parametric evaluation revealed the potential of different MSC-EV preparations to recruit endothelial cells and to detect an enhanced recruitment capacity of highly metastatic PC3-derived EVs (PC3-EVs) compared to low-metastatic LNCaP-EVs in a tumour cell-specific manner.

**Conclusions:**

Overall, this agarose spot migration assay may offer a diversity of measurements and migration settings not provided by classical migration assays and reveal its potential use in the EV field in two different contexts with recruitment in common: regeneration and cancer metastasis.

**Supplementary Information:**

The online version contains supplementary material available at 10.1186/s12915-023-01729-5.

## Background

Extracellular vesicles (EVs) have shown to exert different functions in multiple pathophysiological situations. One of these functions is the capability of EVs to recruit effector cells, as already described in embryo implantation [1, 2], regenerative medicine [3], and cancer metastasis [4, 5]. As studies on the recruiting capacity of EVs from different origins and their interaction with different cell types may help to understand more in-depth EV pathophysiology, different assays to test EV-induced cell migration have been developed [6]. Unfortunately, most of these assays, such as the wound healing assay or the cell exclusion zone assay, fail to reproduce the situation in which EVs are locally administered using biomaterial matrixes, matrigels, or scaffolds [3, 7–10] or found in pathological situations, such as in the pre-metastatic niche (PMN) [4, 5]. Also, classical migration tests only indicate cell movement in the presence of a stimulus and not directional cell recruitment by the chemoattractants. In addition, most techniques demand a cell monolayer and are not adequate when the cell type of study requires coating surfaces, as manual scratches or the addition of physical barriers can disturb the coating [11].

To better approach these different situations, we adapted the agarose spot migration assay to evaluate cellular migration induced by EVs. While widely used to evaluate cytokines as chemoattractant stimuli [12–14], the agarose spot assay has been marginally used to evaluate the recruitment potential of EVs [2] or secretomes [15]. Furthermore, all these studies reported limited data (Additional file 1: Table S1) and did not underline nor exploit the advantages of the EV-agarose-spot assay compared to other well-established migration assays. Indeed, the EV-agarose-spot migration assay may allow multiparametric determinations permitting more robust comparisons between experimental conditions (Table 1).

**Table 1:** Comparison of the agarose spot migration assay with other commonly used migration assays

	Agarose spot migration assay	Trans-well migration assay	Wound healing assay or similar	Capillary chamber assay
Chemotaxis	Yes	Yes	No	Yes
Stable gradient of the stimuli	Yes	No	No	No
Measurements	Number of cells Migration area and distance Velocity directionality	Number of cells	Migration area	Number of cells Migration area
Live imaging	Yes	No	Yes	Yes
Type of analysis	Endpoint and kinetic	Endpoint	Endpoint and kinetic	Endpoint and kinetic
Multiple stimuli/well	Yes	No	No	Limited

To illustrate this, in this study, we show several parameters that can be reported from an EV-agarose-spot migration assay using different types of EVs as migration stimuli and in two different experimental contexts, including regeneration (using mesenchymal stromal cell derived-EVs (MSC-EVs)) and cancer metastasis (using different prostate cancer cell lines). Both are examples in which the agarose spot migration assay would be a useful approach to study in vitro the EV-rich niche found upon local delivery of MSC-EVs through EV-retention systems or within the pre-metastatic niche (PMN) to recruit tumour cells.

## Results

### EV purification and characterization

Immortalized Wharton’s jelly mesenchymal stromal cell-derived EVs (iWJ-MSC-EVs), PC3-EVs, and LNCaP-EVs were isolated from iWJ-MSC, PC3, and LNCaP conditioned media respectively, by size exclusion chromatography (SEC). The SEC elution profiles showed that iWJ-MSC-EVs were positive for the EV marker CD63 and the MSC marker CD90, and PC3-EVs and LNCaP-EVs were positive for the EV markers CD63 and CD9. In all cases, EVs eluted separated from the protein of the sample, as previously published [16] (Additional file 2: Fig. S1A and Additional file 3: Fig. S2). The cryo-TEM analysis of the pooled iWJ-MSC-EV fractions confirmed the double membrane, round shape, and nanosized isolated vesicles (Additional file 2: Fig. S1B).

EV integrity inside the agarose spots was also confirmed by super-resolution microscopy. To assess this, agarose spots containing whole labelled iWJ-MSC-EVs and agarose spots containing lysate iWJ-MSC-EVs were analysed. Small fluorescent dots were only observed in agarose spots containing iWJ-MSC-EVs, while agarose spots containing lysate EVs showed only diluted unspecific fluorescence (Additional file 4: Fig. S3A). The super-resolution images of the agarose spots containing iWJ-MSC-EVs confirmed the presence and integrity of round-membraned EV (Additional file 4: Fig. S3B).

### Partial spot image analysis at the endpoint

**Fig. 1:**
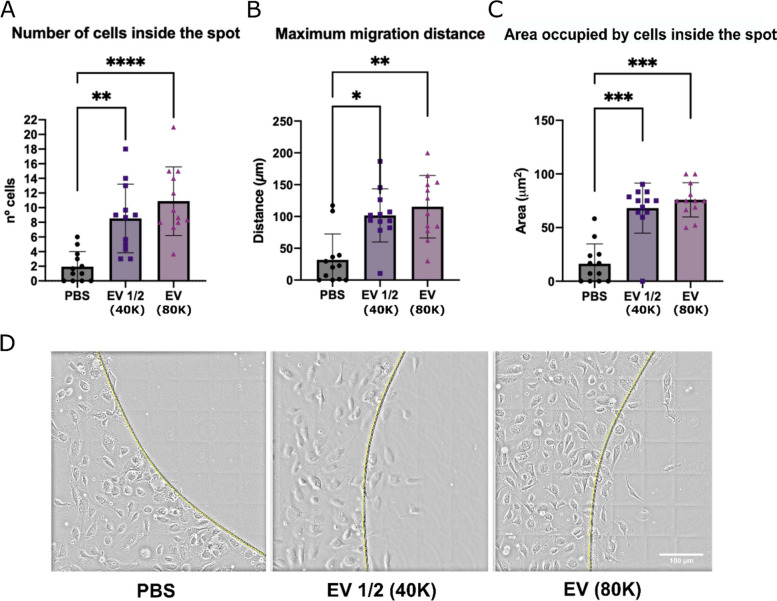
HUVEC recruitment by iWJ-MSC-EV-containing agarose spots: partial spot image analysis (12 h). Quantification of HUVEC’s migration at the final time-point: **A** number of cells inside the spot, **B** maximum migration distance from the spot’s border, and **C** total area occupied by cells inside the spot. **D** Representative images (× 20) of the HUVEC’s recruitment in all conditions. Scale bar is 100 μm. Agarose spot’s border is represented as a yellow dotted line. Data from 4 independent experiments with 3 replicates/condition. The statistical differences are indicated for *p < 0.05, **p < 0.01, ***p < 0.001, and ****p < 0.0001, by a Kruskal–Wallis test with a Dunn’s post hoc analysis

The endpoint images of the agarose spot migration assay allow us to quantitatively analyse cell migration through different and complementary parameters, such as the number of cells that have entered the spot after a given time, the travelled distance of the migrating cells from the spots border, or the area occupied by the cells inside the spot. The analysis of these parameters in the iWJ-MSC-EV-containing agarose spots showed a significant increase in the number of human vein endothelial cells (HUVEC) that had entered the spot in both the half (EV ½, *p* < 0.01) and complete EV dose used (EV, *p* < 0.0001), compared to the PBS-containing agarose spots (Fig. 1A and D). Also, HUVEC’s maximum migration distance in those spots containing iWJ-MSC-EVs was significantly increased in both EV doses (*p* < 0.05 for EV 1/2 and *p* < 0.01 for EV) (Fig. 1B and D). Accordingly, the area occupied by cells inside the spot was also significantly higher in those spots containing iWJ-MSC-EV (*p* < 0.001 for EV 1/2 and EV) (Fig. 1C and D).

### Analysis of live cell migration: video or concatenated images

**Fig. 2:**
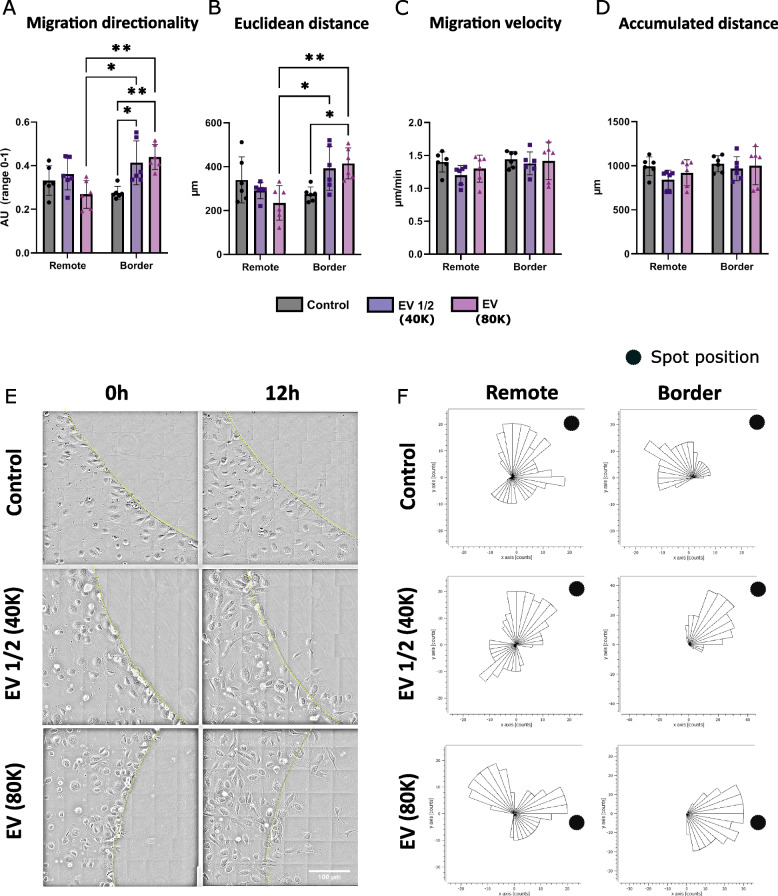
HUVEC recruitment by iWJ-MSC-EVs embedded in agarose spots: live analysis of the active cell migration followed for 12 h. Quantification of the **A** directionality, **B** Euclidean distance, **C** velocity, and **D** accumulated distance of the cell movement. Images were taken every 30 min, tracking 5 border and 5 remote random cells of 6 different spots for each condition. **E** Representative images of the migration assay from each condition at 0 and 12 h. Scale bar is 100 μm. Agarose spot’s border is represented as a yellow dotted line. **F** Corresponding rose plots of each condition of the HUVEC’s movement during the 12 h follow-up. The spot’s position is represented as a black dot. Border (cells localised at the spot’s border) and remote (cells localised far away from the spot) cells positions were determined at time 0. Results from 2 independent experiments with 3 replicates/conditions are shown. Statistically significant differences are represented as *p < 0.05 and **p < 0.01 by a Kruskal–Wallis test with a Dunn’s post hoc analysis

Live imaging of cell migration in the agarose spot migration assay also permits to determine the directionality of the cell movement, migration velocity, accumulated migration distance, and Euclidian distance. The analysis of HUVEC tracking for 12 h in our setting revealed a directional migration only towards the agarose spots containing iWJ-MSC-EVs (*p* < 0.05 for EV 1/2 and *p* < 0.01 for EV) (Fig. 2A and E; Additional file 5: Video 1, Additional file 6: Video 2, and Additional file 7: Video 3). Furthermore, this directionality was only observed in those cells in contact with the spot border. When cells were positioned far away from the spot border (remote area) (Fig. 4), cell movement was random irrespectively of the spot load (Fig. 2A and F). Similar results were observed in terms of Euclidian distance. However, only the higher dose of iWJ-MSC-EVs was able to increase the migration distance of border cells (*p* < 0.05). As before, no differences between conditions were observed in remote localized cells, which behaved identically (Fig. 2B). Finally, no changes in cell velocity (Fig. 2C) or accumulated migration distance of the cells analysed were found (Fig. 2D).

### Whole spot image analysis at the endpoint

The area occupied by cells inside the spot is a parameter that may be of interest when cell size makes cell number quantification difficult. As cell migration may not be uniform across the agarose spots, in this type of quantification, a whole spot image analysis was done to get more robust results and to show another migration measurement option.

**Fig. 3:**
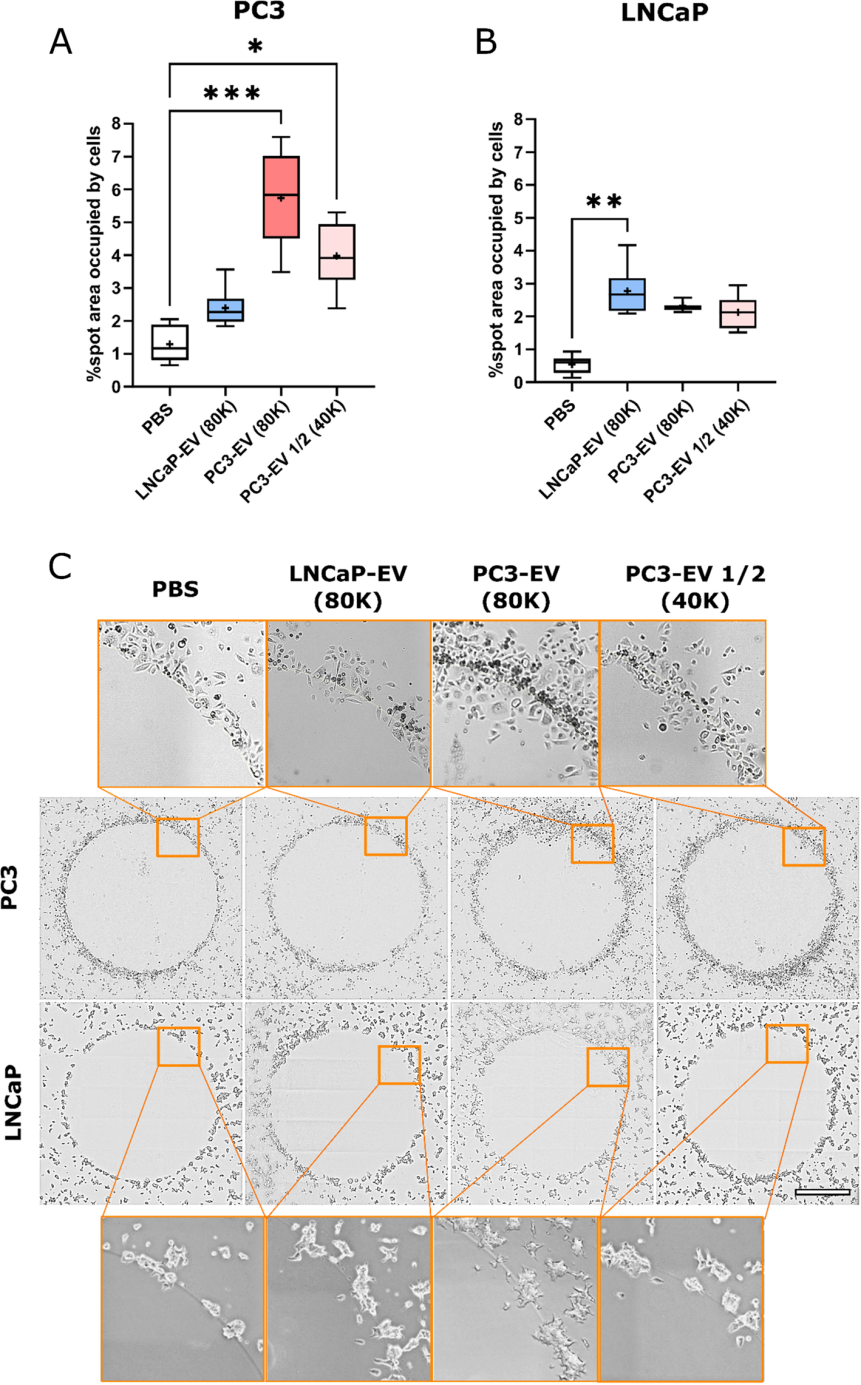
High pro-metastatic PC3 and low pro-metastatic LNCaP cell lines cis/trans recruitment by their own EVs embedded in agarose spots: whole spot image analysis (48 h). **A** Quantification of PC3 migration. **B** Quantification of LNCaP migration. The percentage of spot area occupied by cells was measured at final time-point images (48 h). **C** Representative mosaic pictures (× 20) of the PC3 and LNCaP recruitment of each condition. Data from two independent experiments with 3 replicates/condition. Scale bar is 1 mm. The statistical differences are indicated for *p < 0.05, **p < 0.01, and ***p < 0.001, by a Kruskal–Wallis test with a Dunn’s post hoc analysis

To perform this approach, the high pro-metastatic PC3 and low pro-metastatic LNCaP cell lines were exposed to their own EVs and/or EVs produced by the other cell line. The percentage of the spot area occupied by cells was significantly increased when both cell types were attracted to their EVs (*p* < 0.001 for PC3 and *p* < 0.01 for LNCaP, Fig. 3A and B). Notably, the high pro-metastatic PC3 cells occupied a remarkably higher migration area in response to their EVs (*p* < 0.001), in a dose-dependent manner. More interestingly, PC3 migration capability was less when exposed to the low pro-metastatic LNCaP-EVs (from 6 to 2.3%; *p* < 0.01 by one-to-one Student’s *T* test) suggesting the existence of a specific cis-attracting factor. In contrast, LNCaP cell line migrated similarly to their EVs when exposed to EVs from the highly metastatic PC3 (2.6% vs. 2.3%; n.s.) (Fig. 3).

## Discussion

The role and importance that extracellular vesicles (EVs) play in multiple biological scenarios are increasingly studied. The capacity to recruit effector cells is one of the latest functions described for these nanovesicles. In this context, specific methods such as the adapted agarose spot migration assay can help researchers unveil EV pathophysiology.

The therapeutic effect of mesenchymal stromal cell derived-EVs (MSC-EVs) has been widely demonstrated in different fields [17]. Lately, local administration of MSC-EVs is becoming an increasingly used option to achieve the highest EV retention and therapeutic effect, as in tissue regeneration through pro-regenerative cell recruitment. On the other hand, in cancer metastasis, increasing evidence suggests that cancer EVs play a crucial role in establishing the pre-metastatic niche (PMN) by recruiting primary tumour cells. Yet, little is known about the mechanisms underlying this process. Thus, in both fields, new methodologies to study and better understand the migration processes enhanced by MSC-EVs or cancer EVs are necessary.

Cell migration and invasion are important processes for tissue homeostasis and repair in healthy and pathological contexts [18]. In this context, a variety of in vitro migration assays have already been established to understand migration mechanisms as reviewed in Kramer et al. [6]. However, each technique may not be suitable for each cell type and study setting. Most of these assays, such as the wound healing assay or the cell exclusion zone assay, fail to reproduce the situation in which EVs are released by EV-containing biomaterial structures or in which cancer EVs recruit metastatic cells to the PMN. Also, most techniques demand a cell monolayer and are unsuitable when a coating surface is required by the cell type [11]. Furthermore, only one condition or stimuli/experiment per plate can be analysed in these settings. Other well-established migration assays, such as the Boyden, Zigmond, Dunn, or Insall chambers, based on chemotaxis gradients between two separate chambers, which would be more suited to our study situation, can be poorly adapted to investigate cellular migration towards multiple chemoattractants and do not provide any information regarding migration directionality or Euclidean distance travelled by cells.

To face these shortcomings and limitations, we analysed multiple parameters that can be reported from an EV-agarose-spot migration assay which may overcome most of the aforementioned pitfalls. First, the chemoattractants are retained in the porous structure of the agarose. This retention similarly reproduces the way EVs are delivered by local EV administration structures or the way cancer EVs are found in the PMN. It also enables to follow not only cell movement but also EV-recruitment capacity and specificity. Second, all spot conditions are placed on the same surface/well, with equal culture conditions diminishing the variability of basal parameters between experiments or replicates. The cells responding towards the stimuli are also the same for each condition, which is translated again in less uncontrolled variables and increased robustness of the results. Third, the agarose spot migration assay is compatible with cells demanding a coating to grow attached, as no surface modification or alteration (i.e. scratches) are needed. In our setting, fibronectin coating was applied to the whole surface before the agarose spot, and no inconveniences were observed related to agarose spot attachment or HUVEC adhesion. Fourth, this assay offers a wide range of parameters and variables to analyse cell migration.

In line to classical migration analyses, the distance, the area occupied by cells inside the spot, and the number of migrated cells at endpoint, as measured in our settings, lead to a consistent and complementary analysis of the migration assay [13], confirming the objectiveness of the agarose spot migration assay. In our case, all the parameters analysed indicated an increased migration in those spots containing both doses of iWJ-MSC-EV. Of note, it is important to consider the differential information provided by each individual parameter, all together contributing to the robustness of the EV-agarose-spot assay.

Alternatively, unlike the well-established trans-well migration assay [6], the agarose spot migration assay also allows live tracking when performed in a microscope incubation system, under constant temperature (37 °C) and CO_2_ concentration (5%). After time-lapse image acquisition, pictures can be concatenated or built as a video and analysed using cell tracking software. This kind of analysis provides even more robustness to the results, as it enables quantifying additional migration parameters, such as directionality, Euclidian distance, velocity, and accumulated migration distance of the cell movement, that cannot be determined by endpoint images (Table 1). In our experiments, the tracking analysis revealed an increased Euclidian distance in the agarose spots containing iWJ-MSC-EVs and a directional migration only towards those spots. These results were seen for those cells in close contact with the agarose spot’s edge as previously described by Ahmed et al. (13) and Monguió-Tortajada et al. (3), while remote cells moved randomly and without directionality. Thus, probably EVs need to be closely sensed by cells to migrate. The establishment of a concentration gradient in the agarose spot and the presence of chemokines on the EV surface, also previously described [13, 19], could explain these observations.

In a parallel scenario, the metastatic potential of different prostate cancer cell lines has been widely described. PC3 cells have high metastatic capacity compared to the LNCaP cell line [20], and EVs are well recognized as major players allowing cancer cell interactions and microenvironment conditioning [21]. In this context, the EV-agarose-spot migration assay suggests that the highly metastatic PC3 cell line migration is EV-specific, as PC3 cells barely migrated to low-metastatic LNCaP-EVs. Similarly, low metastatic LNCaP cells poorly migrated even when exposed to metastatic PC3-EVs. Vlaeminch and colleagues [21] reported the presence of TGF-β in PC3-EV and TGF-β receptors in PC3 cells and their absence in LNCaP-EV and LNCaP cells respectively. Given the role of TGF- β on the migratory and invasive capabilities of cancer cells [22], this could partially explain the EV-cell-specific interaction revealed by the EV-agarose-spot assay. A fully dedicated manuscript would be required to confirm this or alternative hypothesis [4, 23, 24], but nonetheless the results illustrate the feasibility and applicability of using the EV-agarose-spot assay for studying the mechanisms associated to metastatic cancer.

## Conclusions

The EV-adapted agarose spot migration assay is a simple, low-cost, and versatile technique that enables to study simultaneously various chemotactic responses of cells towards different stimuli. This test can be easily adapted to the EV field to study their chemoattractant ability in a multiparametric analysis and different scenarios. Overall, it offers measurements and migration settings that can be hardly established by current migration assays.

## Methods

All study protocols with human samples were approved by the Clinical Research Ethics Committee of the Germans Trias I Pujol University Hospital (EO-10–016 and EO-12–022) and followed the principles outlined in the Declaration of Helsinki.

### Cell culture

Immortalized Wharton’s jelly-derived MSC (iWJ-MSC) were cultured in T-175 flasks in alpha-minimum essential medium (alpha-MEM; Sigma Aldrich, St Louis, MO, USA), with 10% of heat-inactivated fetal bovine serum (FBS; Life Technologies, Carlsbad, CA), 0.02 mM of L-glutamine and 1% of penicillin–streptomycin (P/S; Gibco Invitrogen Corp); at 5% of CO_2_ and 37 °C.

Human umbilical vein endothelial cells (HUVEC) were cultured in previously coated 6 well plates with 0.5% of gelatin A (Sigma Aldrich) in endothelial growth media (EGM-2) (Lonza, Basel, Switzerland); at 5% of CO_2_ and 37 °C.

Metastatic prostate cancer cell lines PC3 (highly metastatic) and LNCaP (low metastatic) were cultured in T-175 flasks in Ha’s F-12 K (Kaigh’s) and RPMI Medium (Thermo Fisher Scientific, Waltham, MA, USAG) respectively, with 10% of FBS (Life Technologies, Carlsbad, CA), 0.02 mM of L-glutamine, and 1% of penicillin–streptomycin (P/S; Gibco Invitrogen Corp); at 5% of CO_2_ and 37 °C.

### EV isolation and characterization

iWJ-MSC-EVs, PC3-EVs, and LNCaP-EVs were isolated by size exclusion chromatography (SEC) following the previously published protocol [16, 25]. All cell lines were cultured in a T-175 flask until confluence (10^7^ cells). Then, the medium was replaced by 15 mL of bovine EV-depleted medium, prepared as previously published [16, 25], and cells were cultured for 48 h. Next, the conditioned medium (CM) was collected and sequentially centrifuged at 400 × *g* for 5 min and at 2000 × *g* for 10 min, to exclude cells and cell debris, respectively. The CM was then concentrated (CCM) by a 100-kDa ultrafiltration using an Amicon Ultra centrifugal filter (Millipore, Billerica, MA, USA) at 2000 × *g* for 40 min, to 300 µL. One hundred fifty microliters of the CCM was then loaded into a SEC column, and EV isolation was performed. Sterile 1 × PBS was used as elution buffer, and twenty 100-µL fractions were collected.

Finally, EV-containing fractions were characterized by bead-based flow cytometry as previously described [16, 25], screening for the MSC marker CD90 and the EV markers CD63 and CD9. The protein content was analysed at 280 nm absorbance, using a Nanodrop spectrophotometer (Thermo Fisher Scientific).

#### Cryogenic-electron microscopy

iWJ-MSC-EV integrity, morphology, and size were analysed by cryogenic-electron microscopy (cryo-TEM) using a transmission electron microscope (Joel JEM 2011, Tokyo, Japan), as previously described [16].

#### Super-resolution analysis of EV embedded in agarose

To confirm EV integrity inside the agarose spots, whole or lysate iWJ-MSC-EV were stained and analysed by super-resolution microscopy. Specifically, iWJ-MSC-EV were stained with DPPE-Abberior STAR RED. Briefly, 4 µL of dye were mixed with 1 mL of diluent C. Then, EVs were mixed 1:1 with the diluted dye and incubated for 5 min with periodically mixing. After incubation, EVs were washed 3 times with PBS using an Amicon Ultra 100 kDa filter (Merck Millipore). Finally, lysate EVs were obtained by RIPA (1x) digestion on ice and periodically vortexing.

Super-resolution analysis of EV structure was performed using Abberior Infinity microscope (Gottingen, Germany) equipped with a 60 × /1.42 NA oil immersion STED objective. STED images of EVs stained with DPPE-Abberior STAR RED were acquired 640 nm excitation line and Abberior STAR RED was depleted with a donut-shaped 775-nm pulsed STED laser.

Under these conditions, approx. 40 nm lateral resolution (full-width-at-half-maximum, FWHM) was achieved for Abberior STAR RED acquisition channel (estimated from fluorescent bead calibration measurements). STED images were acquired with following parameters: pinhole size: 1 AU; dwell time: 5 μs/pixel, XY pixel size: 20 nm. Following acquisition, confocal and STED images were thresholded and smoothed (Gaussian blur, radius = 1.0 Sigma) using Fiji (ImageJ distribution) software.

### Agarose spot migration assay

**Fig. 4:**
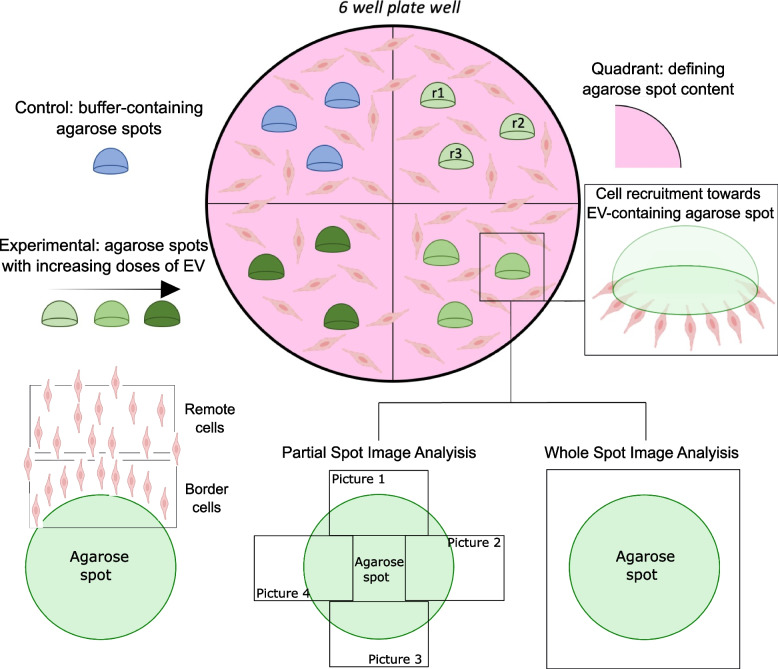
Schematic representation of the agarose spot migration assay performed with HUVEC and iWJ-MSC-EV-containing spots in a 6-well plate well. r1, r2, and r3 depict experimental replicates. The partial spot image analysis and the whole spot image analysis are shown

iWJ-MSC-EVs’ and prostate cancer-EVs’ recruitment capacity was tested in agarose spots, using a modification of a previously published protocol [13, 26]. First, to differentiate between conditions in a same well, the backwards of the wells of a 6-well plate were divided into 4 drawn quadrants. Then, for the HUVEC migration assay with iWJ-MSC-EVs, the wells were coated with 10 µg/mL of fibronectin (Merck Millipore, Darmstadt, Germany) for 30 min at 37 °C. After incubation, the excess of fibronectin was removed and the plate was left to dry as recommended by the manufacturer. Next, low-melting-point agarose (40 °C, Ecogen, Barcelona, Spain) was mixed (1:1) with either PBS (used as a buffer control) or iWJ-MSC-EVs coming from 40,000 (EV ½) or 80,000 (EV) producing cells, and spots of 5 μL from each mix were placed into the corresponding quadrant of the well and incubated for 15 min at 4 °C for agarose solidification and attachment to the well surface. Finally, 100,000 HUVEC were seeded into each well with 2 mL of EGM-2 complete medium (2% FBS) (Fig. 4).

For the prostate cancer cells’ migration assay,the same protocol was followed, but in this case, no fibronectin coating was needed and low-melting-point agarose (40 °C, Ecogen, Barcelona, Spain) was mixed (1:1) with either PBS, PC3-EVs coming from 40,000 ( PC3-EV ½) or 80,000 (PC3-EV) producing cells or LNCaP-EVs coming from 80,000 producing cells. Furthermore, a higher number of LNCaP cells were seeded (300.000) per well due to its small size.

To analyse HUVEC’s live migration in response to iWJ-MSC-EV-containing agarose spots, the plate was incubated under controlled temperature (37 °C) and CO_2_ levels in the Axioskop Z1 microscope (Zeiss) incubation system for 12 h. 7 × 7 mosaic images at × 20 objective were taken every 30 min. Specifically, random localizations of the spot’s border of each condition, determined at time 0, were captured. Finally, all the pictures were compiled in videos, and 5 cells next to the spot’s border and 5 other cells far away from it (remote position) were followed using the Manual Tracking plugin Fiji software (Image J 1.54w, NIH, USA) and the Chemotaxis and Migration tool software (v.2.0 Ibidi, Inc.) to quantify the Euclidian distance (the distance between two points defined as the square root of the sum of the squares of the differences between the corresponding coordinates), the directionality and velocity of the cell movement, and the accumulated travelled distance of the cells analysed.

Additionally, endpoint images (12 h follow-up) showing only a part of the agarose spot were used to measure the area occupied by cells inside the spot, the distance of the 3 most distant cells from the agarose spot’s edge, and the number of cells inside the spot. Specifically, the distance travelled by all cells that had entered the spot from the spot’s edge was measured using the line selection tool of Image J software (Image J 1.54w, NIH, USA). Then, the average value of the 3 most distant cells was used as an indication of the maximum migration distance.

To show other measurement options of the agarose spot migration assay, for the prostate cancer cells’ migration assays a whole spot image analysis of endpoint images (48 h follow-up) was performed. For this approach, mosaic images (× 20) were taken under the inverted microscope Leica DMI6000 B (Leica microsystems, Wetzlar, Germany) and migration area (% area occupied by cells inside the spot) was determined using Image J software (Image J 1.54w, NIH, USA).

### Statistics

All data are shown as mean with SD and all test are two-tailed. The normality of data was checked by Shapiro–Wilk test before applying the appropriate statistical analysis using the GraphPad Prism software (9.0 version). Only significant statistical differences are shown (*p* < 0.05).

### Supplementary Information

**Additional file 1**:** Table S1.** Publications that have used the agarose spot migration assay before, with their migration parameters analysed and stimuli used in the assays [27–37].

**Additional file 2**: **Fig. S1.** iWJ-MSC-EVs were isolated by SEC and characterized by bead-based flow cytometry and cryo-TEM. (A) Representative SEC elution profile of iWJ-MSC-EVs, positive for the EV and MSC makers CD63 and CD90, respectively. Protein elution was measured at 280nm absorbance by nanodrop and occurred later, separated from EV fractions. (B) Representative picture of iWJ-MSC-EV taken by cryo-TEM, which confirmed the presence of round-shaped and double membraned nanovesicles of 50-300nm. Scale bar is 500nm.

**Additional file 3**: **Fig. S2.** LNCaP-EVs and PC3-EVs were isolated by SEC and characterized by bead-based flow cytometry. Representative SEC elution profiles of (A) LNCaP-EV and (B) PC3-EV, both positive for the EV makers CD63 and CD9. Protein elution was measured at 280nm absorbance by nanodrop and occurred later, separated from EV fractions.

**Additional file 4**: **Fig. S3.** Fluorescence microscopy images of agarose spots containing iWJ-MSC-EV. (A) Two Z-stack planes of the agarose spots containing labelled iWJ-MSC-EV and labelled plus lysate iWJ-MSC-EV are shown (bar 10 μm). (B) Super-resolution images of grouped and individual iWJ-MSC-EV (bar 500 nm).

**Additional file 5**: **Video 1.** HUVEC migration time lapse in an agarose spot containing PBS.

**Additional file 6**: **Video 2.** HUVEC migration time lapse in an agarose spot containing iWJ-MSC-EV 1/2 (40K).

**Additional file 7**: **Video 3.** HUVEC migration time lapse in an agarose spot containing iWJ-MSC-EV (80K).

## Data Availability

The datasets used and/or analysed during the current study are available from the corresponding author on reasonable request.

## Abbreviations

EVs: Extracellular vesicles
HUVEC: Human umbilical vein endothelial cells
MSC-EVs: Mesenchymal stromal cell-derived extracellular vesicles
iWJ-MSC-EVs: Immortalized Wharton’s jelly mesenchymal stromal cell-derived extracellular vesicles
TGF-β: Tumour growth factor-beta
PMN: Pre-metastatic niche
SEC: Size exclusion chromatography
FBS: Fetal bovine serum
