# Supplementary material for: Agarose spot migration assay to measure the chemoattractant potential of extracellular vesicles: applications in regenerative medicine and cancer metastasis
Source: BMC Biol. 2023 Oct 26;21:236. doi: 10.1186/s12915-023-01729-5 (PMC10605981; doi:10.1186/s12915-023-01729-5)
Supplement: Supplementary file 1 — Additional file 1: Table S1. Publications that have used the agarose spot migration assay before, with their migration parameters analysed and stimuli used in the assays [27–37]. [file 12915_2023_1729_MOESM1_ESM.docx]

| References | Migration parameter measured | | Stimuli inside the spot |
| --- | --- | --- | --- |
| Wiggins and Rappoport, 2010[26] | | Number of migrated cells  Migration distance | DMSO and EGF |
| Vinader et al., 2011[27] | | Average number of cells per field | Cytokines |
| Smith et al., 2012[28] | | Number of migrated cells | Dentine chip, dentine powder, ECM extracts |
| Szatmary et al., 2014[29] | | Gradient inside the spot of the chemoattractant | Cytokines |
| Ahmed et al., 2017[13] | | Gradient inside the spot of the chemoattractant  Relative number of migrated cells Directionality and chemotaxis | Chemokines |
| Aquino-Martínez et al., 2017[30] | | Number of migrated cells  Area occupied by cells inside the spot | CaSO_4_ solution |
| Loch et al., 2018[31] | | Number of migrated cells | INFɣ |
| Calle et al., 2018[14] | | Migration distance | Cytokines |
| Aquino-Martínez et al., 2019[32] | | Number of migrated cells | Conditioned medium or calcium |
| Calle et al., 2019[12] | | Migration distance | Cytokines |
| Hwang et al., 2020[33] | | Analysis of cells migration range per hour in agarose spots | Histamine |
| Calle et al., 2021a[2] | | Migration distance | EV and soluble proteins |
| Calle et al., 2021 b[15] | | Maximum Euclidean distance  Velocity of migration  Center of mass length | Cytokines and secretome |
| Monguió-Tortajada et al., 2021[3] | | Directionality  Migration Euclidean distance | MSC-EV |
| Hwang et al., 2021[34] | | Analysis of cells migration range per hour in agarose spots | Spinophilin |
| He et al., 2022[35] | | Number of migrated cells | LPS + BAY |
| Binlateh et al., 2022[36] | | Area occupied by cells inside the spot | No stimuli inside the spot. Cells with different treatments were tested |
| Clos-Sansalvador et al., 2022[37] | | Maximum migration distance  Area occupied by cells inside the spot | MSC-EV |
