# Supplementary figures and images for: Agarose spot migration assay to measure the chemoattractant potential of extracellular vesicles: applications in regenerative medicine and cancer metastasis
Source: BMC Biol. 2023 Oct 26;21:236. doi: 10.1186/s12915-023-01729-5 (PMC10605981; doi:10.1186/s12915-023-01729-5)

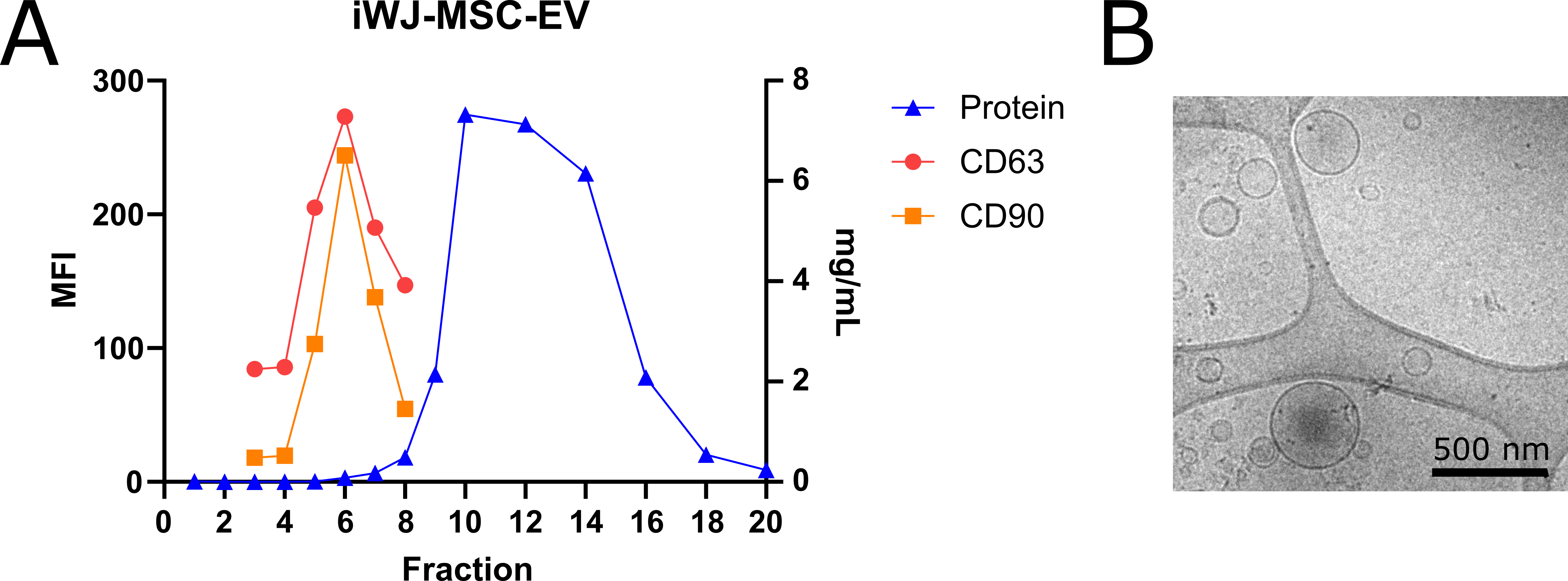

Supplement: Supplementary file 2 — Additional file 2: Fig. S1. iWJ-MSC-EVs were isolated by SEC and characterized by bead-based flow cytometry and cryo-TEM. (A) Representative SEC elution profile of iWJ-MSC-EVs, positive for the EV and MSC makers CD63 and CD90, respectively. Protein elution was measured at 280nm absorbance by nanodrop and occurred later, separated from EV fractions. (B) Representative picture of iWJ-MSC-EV taken by cryo-TEM, which confirmed the presence of round-shaped and double membraned nanovesicles of 50-300nm. Scale bar is 500nm. [file 12915_2023_1729_MOESM2_ESM.png]

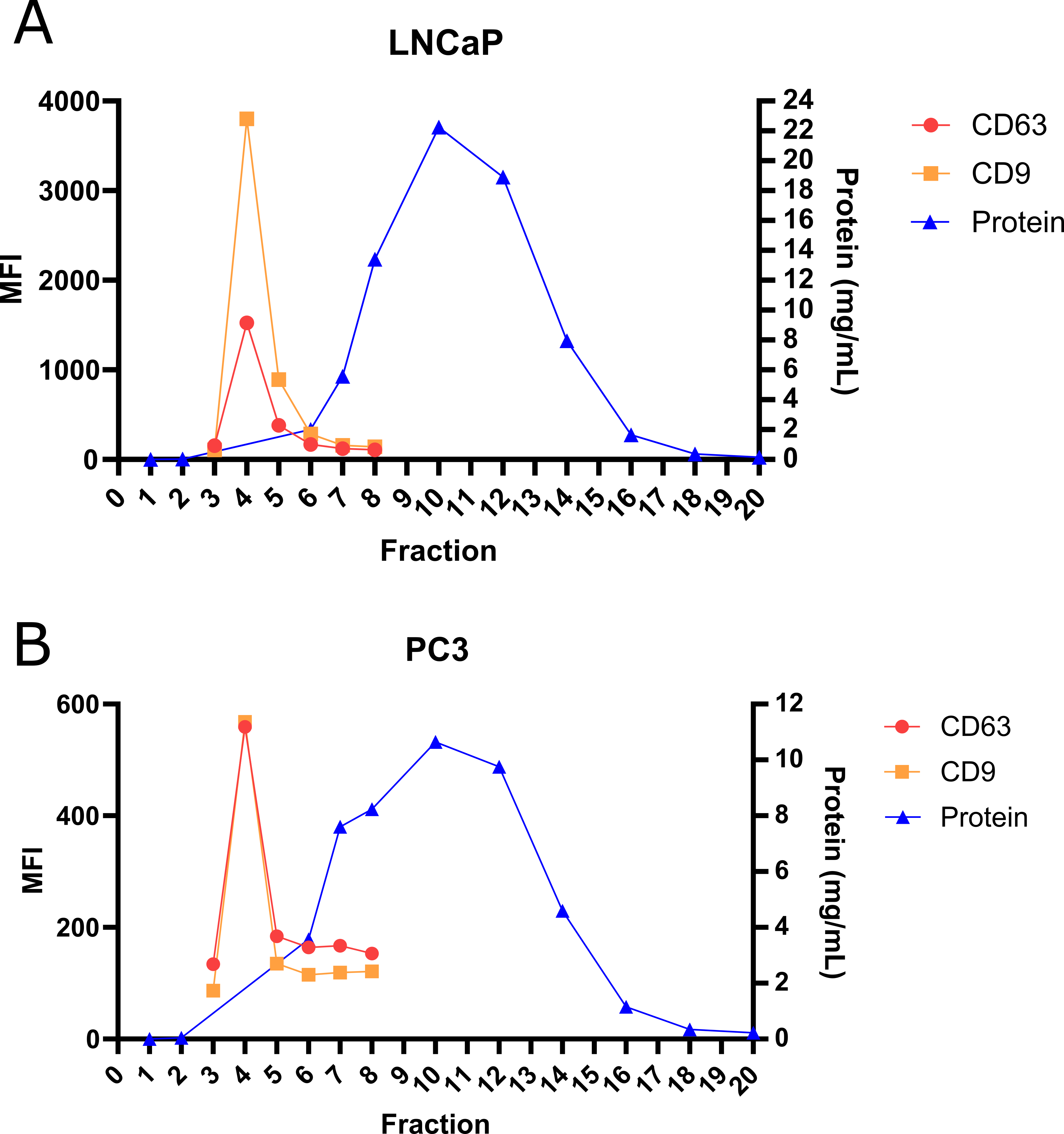

Supplement: Supplementary file 3 — Additional file 3: Fig. S2. LNCaP-EVs and PC3-EVs were isolated by SEC and characterized by bead-based flow cytometry. Representative SEC elution profiles of (A) LNCaP-EV and (B) PC3-EV, both positive for the EV makers CD63 and CD9. Protein elution was measured at 280nm absorbance by nanodrop and occurred later, separated from EV fractions. [file 12915_2023_1729_MOESM3_ESM.png]

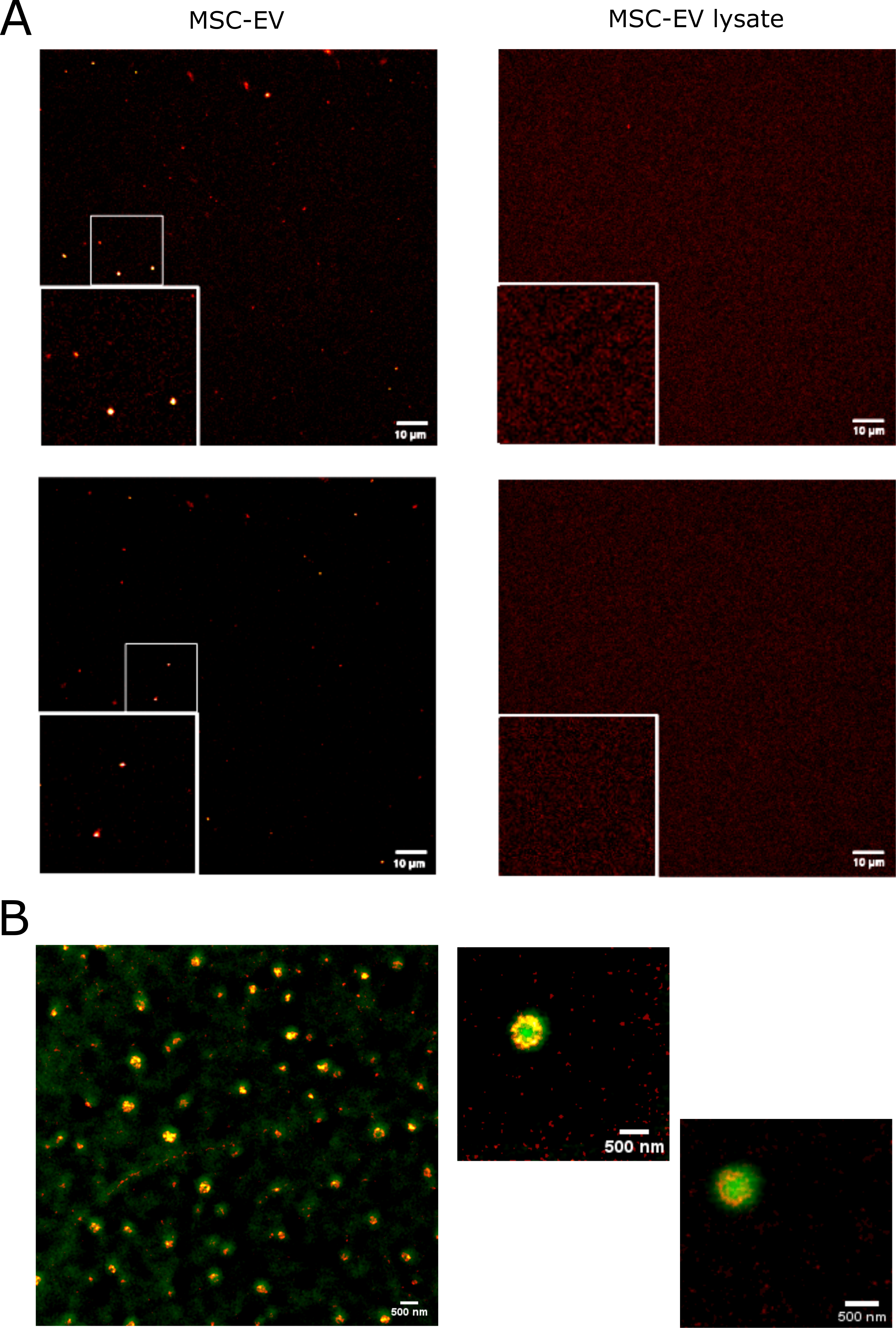

Supplement: Supplementary file 4 — Additional file 4: Fig. S3. Fluorescence microscopy images of agarose spots containing iWJ-MSC-EV. (A) Two Z-stack planes of the agarose spots containing labelled iWJ-MSC-EV and labelled plus lysate iWJ-MSC-EV are shown (bar 10 μm). (B) Super-resolution images of grouped and individual iWJ-MSC-EV (bar 500 nm). [file 12915_2023_1729_MOESM4_ESM.png]
